# Virulence factors and quorum sensing as targets of new therapeutic options by plant-derived compounds against bacterial infections caused by human and animal pathogens

**DOI:** 10.14202/vetworld.2023.1346-1355

**Published:** 2023-06-14

**Authors:** Warangkana Kitpipit, C. Norman Scholfield, Suthinee Sangkanu, Veeranoot Nissapatorn, Maria de Lourdes Pereira, Alok K. Paul, Watcharapong Mitsuwan

**Affiliations:** 1 Akkhraratchakumari Veterinary College, Walailak University, Nakhon Si Thammarat, 80160, Thailand; 2 One Health Research Center, Walailak University, Nakhon Si Thammarat, 80160, Thailand; 3 Food Technology and Innovation Center of Excellence, Walailak University, Nakhon Si Thammarat, 80160, Thailand; 4 School of Allied Health Sciences, Southeast Asia Water Team, World Union for Herbal Drug Discovery, and Research Excellence Center for Innovation and Health Products, Walailak University, Nakhon Si Thammarat, 80160, Thailand; 5 Department of Medical Sciences, CICECO-Aveiro Institute of Materials, University of Aveiro, Aveiro, Portugal; 6 School of Pharmacy and Pharmacology, University of Tasmania, Hobart, TAS 7001, Australia; 7 Center of Excellence in Innovation of Essential Oil and Bioactive Compounds, Walailak University, Nakhon Si Thammarat, 80160, Thailand

**Keywords:** antibiotic resistance, pathogens, phytochemicals, quorum sensing system, virulence factors

## Abstract

The emergence of antibiotic-resistant bacteria and hospital-acquired bacterial infection has become rampant due to antibiotic overuse. Virulence factors are secondary to bacterial growth and are important in their pathogenesis, and therefore, new antimicrobial therapies to inhibit bacterial virulence factors are becoming important strategies against antibiotic resistance. Here, we focus on anti-virulence factors that act through anti-quorum sensing and the subsequent clearance of bacteria by antimicrobial compounds, especially active herbal extracts. These quorum sensing systems are based on toxins, biofilms, and efflux pumps, and bioactive compounds isolated from medicinal plants can treat bacterial virulence pathologies. Ideally, bacterial virulence factors are secondary growth factors of bacteria. Hence, inhibition of bacterial virulence factors could reduce bacterial pathogenesis. Furthermore, anti-virulence factors from herbal compounds can be developed as novel treatments for bacterial infection. Therefore, this narrative review aims to discuss bacterial virulence factors acting through quorum sensing systems that are preserved as targets for treating bacterial infection by plant-derived compounds.

## Introduction

The emergence of infections caused by pathogenic antibiotic*-*resistant bacteria in the clinical environment is of global concern, as highlighted by the World Health Organization [1]. Antibiotic resistance now includes drugs that hitherto provided the “last line of defense,” resulting in an alarming increase in global morbidity and mortality and further stresses health provision [2]. Antibiotics target bacterial growth factors involving cell wall, protein, and nucleic acid biosynthesis. Overuse or inappropriate application of antibiotics further escalates antibiotic-induced resistance. Bacterial infection follows the failure of host immune responses to remove these pathogens [3].

Virulence factors also assist bacterial invasion and adhesion of tissues, further evading host immunity and bacterial pathogenesis. Bacterial virulence factors are secondary growth factors regulated by the quorum sensing system and cell communication. Hence, virulence factor inhibition could reduce bacterial pathogenesis, giving host immunity more time to clear the remaining invaders. Thus, anti-virulence factor drugs may provide an effective adjunct to antibiotic treatment. Because plants suffer continual microbial onslaught, antimicrobial phytochemicals isolated from medicinal plants may yield a particularly rich source of anti-virulence factors. In addition, the compounds enhance the activities of host immune cells to remove the microorganism. The compounds act as resistant modifying agents. These agents can be combined with antibiotics to inhibit bacterial resistance mechanisms and restore antibiotic and immune activity [2].

This narrative review provides an overview of virulence factors that target the wider veterinarian community. We aim to show that the use of herbal compounds with antimicrobial activity must be analyzed and propose a strategy for further study in this field, both *in vitro* and *in vivo*.

## Virulence Factors

**Figure-1:**
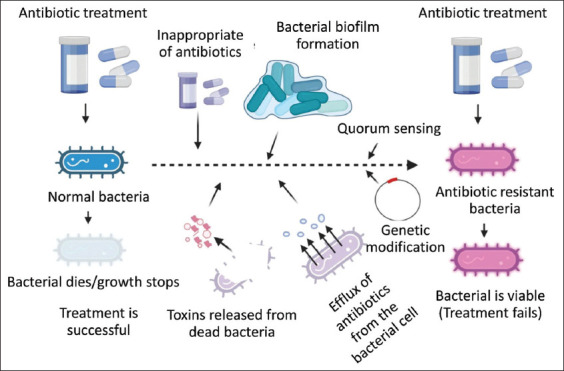
Induction of antibiotic resistance by some virulence factors of the bacteria such as biofilm, efflux pumps, and genetic modification, resulting in the resistance to the antibiotics. [Source: Biorender.com. This figure is prepared by AKP].

Virulence factors are an important strategy for bacteria to infect a host, culminating in severe pathology [4]. Virulence factors are secondary growth systems of the bacterium that maintain bacterial survival and enhance its potential to cause disease. Virulence factors are secreted molecules that assist the microorganism in adhesion and invasion [5]. Virulence factors contain bacterial toxins, biofilms, cell surface proteins, enzymes, capsules, and pili. Antibiotic resistance is caused by the incorrect administration and usage of antibiotics (Figure-1). However, some pathogens express the bacterial virulence factors that possess and enhance antibiotic resistance by several mechanisms (Figure-1).

## Bacterial Toxin

Bacterial toxins are poisonous substances produced within the microorganism. The toxins are key virulence factors in some bacteria, such as *Clostridium botulinum*. Bacterial toxins are categorized as endotoxins and exotoxins. In Gram-negative bacteria, the endotoxin (e.g., lipopolysaccharide) can cause clinical problems with fever, change in blood pressure, inflammatory response, life-threatening shock, and other fatal conditions. The exotoxin contains various types of protein toxins and bacterial enzymes secreted by the bacteria. *Clostridium botulinum* and related species produce a neurotoxic protein called botulinum toxin [6]. *Clostridium difficile*, an anaerobic pathogenic bacterium that forms an endospore, causes human enteric infection related to toxin-mediated infection (toxic shock syndrome). The pathogen produces toxins named TcdA and TcdB that cause disruption of the cytoskeleton and tight junctions of the intestinal epithelium [7]. Diphtheria toxin produced by *Corynebacterium diphtheriae* is extraordinarily potent. This toxin enters the cytoplasm and inhibits protein synthesis to cause disease [7]. Pneumolysin is a cytoplasmic toxin that acts as a cholesterol-dependent cytotoxin by binding to cholesterol in the host cell membrane. The toxin is produced by *Streptococcus pneumoniae*. Pneumolysin is released by autolytic cells during the stationary phase of bacterial growth [8]. The mechanism of action of pneumolysin is thought to follow two stages. The binding of the monomeric toxin to the target cell membrane occurs in the first stage. The next stage is lateral movement and oligomerization of the monomers, resulting in the formation of a large pore and the subsequent leakage of intracellular components and influx of water into the cell, leading to cell lysis [8].

## Biofilm

**Figure-2:**
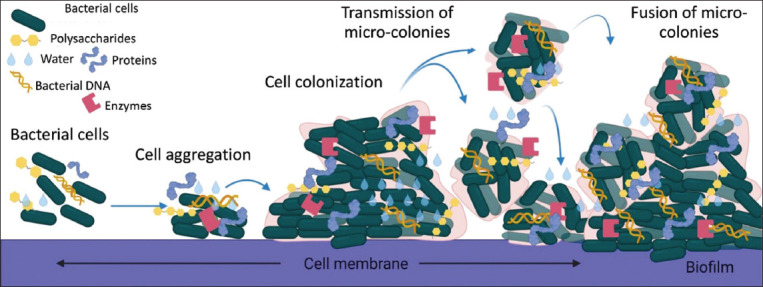
Formation of biofilm of the bacterium. The initial step of biofilm is associated with the attachment between the cell and the surface, followed by adhesion to the surface. Then, bacterial cells produce the exopolysaccharide that consists of the matrix and maturation. Cells assume a planktonic state and can thereby form biofilm in other settings, called detachment. [Source: Biorender.com. This figure is prepared by AKP].

A bacterial biofilm is formed by densely packed communities of bacterial cells embedded within a polymeric substance. The cells within the biofilm produce substances, including polysaccharides, proteins, lipids, and DNA [9]. Biofilm formation consists of three main steps. The initial stage of the biofilm is associated with cell surface attachment, followed by surface adherence (Figure-2). Then, the bacterial cells produce the exopolysaccharide that comprises the matrix and mature from minute colonies into clusters of many layers of cells. Finally, the cells become planktonic and lead to the formation of a biofilm called detachment. Several diverse methods can result in detachment, including erosion, sloughing, or the active release of cells. Detached cells may later colonize new surfaces and begin the production of biofilms elsewhere. [10]. Bacterial biofilms are regulated by the bacterial quorum sensing system [11]. Quorum sensing is a cell-to-cell communication process in bacteria that coincide with gene expression in response to cell densities. Quorum sensing regulates bacterial virulence factors by producing autoinducers [11]. Different bacterial species produce a variety of autoinducers, but acyl-homoserine lactones (AHLs) are the most well-known form. Bacterial biofilms play a potential role in the pathogenesis of bacterial infections. In addition, biofilm formation is a crucial strategic model in host immune evasion that includes phagocytosis [12]. Interestingly, biofilms are associated with antibiotic resistance due to various tolerance mechanisms. Failure in treating *Pseudomonas aeruginosa* infection was reported due to the formation of strong biofilms [13].

## Capsule

The bacterial capsule consists of polysaccharide layers outside the bacterial cells. The capsule is considered a key virulence factor in some microorganisms, including *S. pneumoniae* [14] and *Klebsiella pneumoniae* [15]. *Streptococcus pneumoniae* can be characterized into more than 90 serotypes according to the composition of its polysaccharide capsule [16]. Capsule formation, one of the crucial pathogenic factors, has been reported to be associated with invasive infection or disease. In addition, the polysaccharide capsule of *S. pneumoniae* plays a role in phagocytosis by preventing complement disposition [17]. In *S. pneumoniae*, the transparent strains are more efficient in colonizing the mucosal surface of the nasopharynx. Conversely, opaque strains are more virulent in systemic infections [18]. In *K. pneumoniae*, regulator mutants showed various colonization defects in a murine pneumonia model [19].

## Efflux Pumps

**Figure-3:**
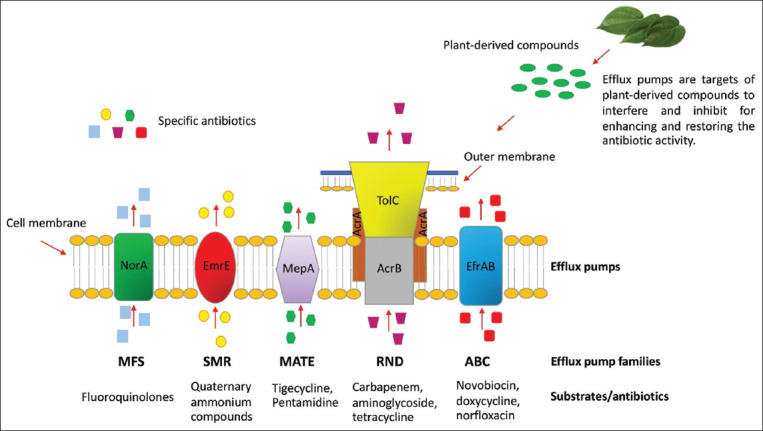
Families of bacterial efflux pumps. Pumps are transport proteins that act in the extrusion of antibiotics from cells to the external environment. There are five families including principal facilitator MFS, MATE, RND, SMR, and ABC. MFS: Major facilitator superfamily, MATE: Multidrug and toxic efflux, RND: Resistance-nodulation-division, SMR: Small multidrug resistance, ABC: ATP-binding cassette. Moreover, Efflux pumps are targets of plant-derived compounds to interfere and inhibit for enhancing and restoring the antibiotic activity. [Source: This figure is prepared by WM, WK, and SS].

Bacterial efflux pumps are carrier proteins that expel toxic substances from cells to the external environment. The pumps have been detected in Gram-positive and Gram-negative bacteria [20]. There are five families of bacterial efflux pumps, including ATP-binding cassette, main facilitator 2, multidrug and toxic efflux, resistance-nodulation-division (RND), and small multidrug resistance (MDR) [21] (Figure-3). The pumps are associated with MDR [22] in many species of pathogenic bacteria, including *Acinetobacter baumannii*, *Escherichia coli*, *P. aeruginosa*, *K. pneumoniae*, and *Staphylococcus aureus* [23]. In *K. pneumoniae*, RNDtype efflux pumps, including AcrAB, OqxAB, EefAB, KexD, KexEF, and KexC, play important role in MDR phenotypes [24]. In addition, MexAB-OprM and MexXY-OprM, members of the RND family, have been reported as powerful multidrug efflux pumps in *P. aeruginosa* [25]. AcrAB-TolC in *E. coli*, AdeFGH in *A. baumannii*, AcrD in *Salmonella enterica*, and MexAB-OprM in *P. aeruginosa* play important roles in biofilm formation [23].

## Quorum Sensing System

**Figure-4:**
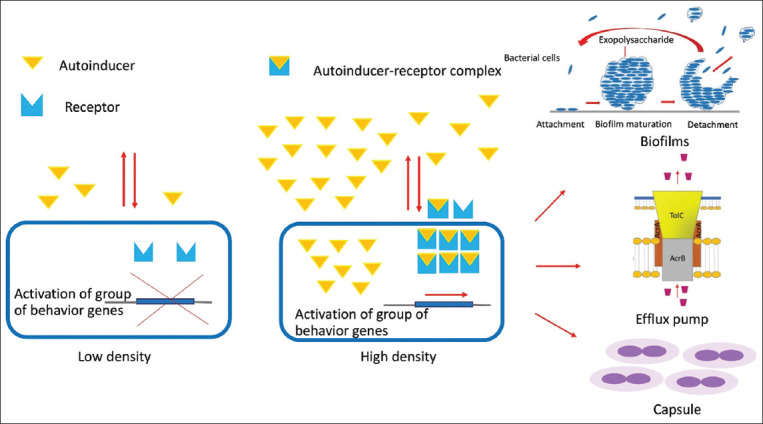
Quorum sensing which cell-to-cell communication depends on the cell density. Bacteria produce and secrete autoinducers, extracellular signaling molecules that bind to receptors to be autoinducer-receptor complex that activates the expressions of the virulence factors such as biofilms, efflux pumps, and capsule. [Source: This figure is prepared by WM, WK, and SS].

The quorum sensing system is a well-known communication system between bacterial cells, depending on cell density (Figure-4). The bacterial quorum sensing system regulates phenotype expression, including virulence factors, for example, biofilm formation, sporulation, toxin production, and swarming. Quorum sensing is regulated by extracellular signaling molecules called autoinducers. Gram-negative bacteria produce *N*-acyl homoserine lactones, whereas Gram-positive bacteria use autoinducing peptides as autoinducers. The expression of the system involved in bacterial survival and virulence traits leads to increased resistance to the host’s immune system and antibiotics [26].

*Chromobacterium violaceum* is a common biomonitoring strain focusing on bioactive materials using quorum sensing actions [26]. Violacein is a purple pigment regulated by the quorum sensing system of *C*. *violaceum* [27]. Violacein is produced under the control of the homologous system LuxR-LuxI, CviR-CviI, and the cognate molecule *N*-hexanoyl-L-homoserine lactone [28]. *Pseudomonas aeruginosa* is a major cause of hospital-acquired bacterial infections. The Gram-negative bacterium is one of the most important pulmonary pathogens in patients with cystic fibrosis [29]. Swarming of *P. aeruginosa* is a multicellular phenomenon that involves coordination and fast movement. Swarming is regulated by the quorum sensing system of *P. aeruginosa*. Furthermore, *P. aeruginosa* with intrinsic resistance to many antibiotics is much more pronounced when pathogenic bacteria are detected in biofilms [10]. The pathogen has four hierarchically connected quorum sensing systems: *las*, *rhl*, *pqs*, and *iqs* for communication between species, which encode 3-oxo-C12-HSL, C4-HSL, 2-alkyl4-qionolones, and 2-(2-hydroxyphenyl)-thiazole-4-carbaldehyde, respectively. The concentrations of autoinducer molecules depend on the growth of the bacterial population and the activated induction of transcriptional regulators [30]. In *S. pneumoniae*, quorum sensing, including biofilm production and autoinducer sensing, is controlled by bacterial virulence factors, called secreted proteins. Rgg/small hydrophobic peptide (SHP) quorum sensing systems are widespread in streptococci, including *S. pneumoniae* [30]. Small hydrophobic peptide acts as an autoinducer of *S. pneumoniae* through Rgg. Rgg/SHP complexes activate binding to the promoter through transcription, resulting in biofilm formation [31]. Consequently, pathogens can combat host immune responses by upregulating virulence genes [32].

## Anti-Virulence Activity

Antibiotic resistance is a major public health concern worldwide. The widespread use of antimicrobial agents is the main driving force behind the evolution of antibiotic resistance. Most antibiotics against pathogens inhibit important bacterial growth factors, including proteins, DNA, RNA, and cell wall components. Hence, bacteria develop mechanisms to survive exposure to antibiotics. *Pseudomonas aeruginosa* cells within biofilms have been documented to be more resistant than planktonic cells [33]. Furthermore, the cells within biofilms make conditions more favorable for bacterial persistence [34]. Hence, the growth rate of bacterial cells within a biofilm is low, resulting in tolerance to beta-lactam antibiotics that inhibit bacterial cell wall synthesis [35]. Therefore, inhibition of the bacterial virulence factors may be an alternative strategy to overcome this problem.

Anti-virulence drugs can be used for treating infections caused by antibiotic-resistant pathogens. Bioactive compounds isolated from medicinal plants can be used to treat infection by inhibiting bacterial virulence factors. Bacterial virulence factors are secondary growth factors of bacteria. Hence, inhibition of bacterial virulence factors by drugs or compounds could reduce bacterial pathogenesis. As shown in Table-1 [36–48], an acid extract of fresh garlic attenuated the virulence factors of *P. aeruginosa*, including biofilms, by inhibiting quorum sensing in the pathogen without inhibiting growth [36]. A *Ginkgo biloba* extract significantly reduced biofilm formation in *E. coli* O157: H7 by repressing the curli and prophage genes in the bacteria. The repression resulted in reduced fimbriae production and biofilm formation [37]. Rhodomyrtone, isolated from the leaves of *Rhodomyrtus tomentosa*, inhibits biofilm formation and the establishment of biofilm in *S. pneumoniae* [38]. Rhodomyrtone suppressed arginine deiminase expression [39], inhibiting biofilm maturation in organisms [38]. In addition, the ethanolic extract of *R. tomentosa* leaves inhibited biofilm formation by *Streptococcus pyogenes* through quorum sensing inhibition [49]. Myrtenol decreased MRSA biofilm and virulence factors by suppressing global *sarA*- and *sarA*-regulated virulence genes [40].

**Table-1:** Virulence factors and the inhibition of the virulence factors by the plant extracts or compounds.

Virulence factors	Microorganisms	Activity of the compounds	Reference
Biofilm	*P. aeruginosa*	Fresh garlic acid extract attenuated *P. aeruginosa* virulence factors including biofilm through inhibition of quorum sensing in the pathogen.	[36]
	*E. coli* O157:H7	*Ginkgo biloba* extract significantly reduced the formation of *E. coli* O157:H7 biofilm through repressing of curli and prophage genes in the bacteria. The repression is resulted in reduction of fimbriae production and biofilm reductions.	[37]
	*S. pneumoniae*	Rhodomyrtone inhibited the formation of biofilm and the establishment of *S. pneumoniae* biofilm. Rhodomyrtone suppressed arginine deiminase expression resulted in inhibition of biofilm maturation in the organisms.	[38, 39]
	Methicillin resistant *Staphylococcus aureus*	Myrtenol decreased MRSA biofilm and virulence factors by suppression of *sarA*- global regulator and *sarA*-mediated virulence genes.	[40]
Capsule	*K. pneumonia*	*Fructus mume* extract inhibited the mucoviscosity and the capsular polysaccharides in *K. pneumoniae* through downregulation of *cps* gens.	[41]
	*S. pneumoniae*	Rhodomyrtone inhibited key enzymes and metabolites in *S. pneumoniae* capsule formation. Rhodomyrtone-treated *S. pneumoniae* significantly possessed fewer numbers of capsule in comparison with the control.	[39]
Toxin	*C. difficile*	Carvacrol, a pure compound isolated oregano oil, inhibited *C. difficile* toxin production in through down regulation of toxin genes, *tcdA* and *tcdB*, in the cell.	[42]
	*V. cholerae*	Crude polyphenol extracted from immature apples inhibited *V. cholerae* toxin induced fluid accumulation in mouse.	[43]
	*E. coli* O157:H7	AHU3 inhibited/suppressed the expression of St×2 in *E. coli* O157:H7. The compound also inhibited RecA and prophage genes encoding virulence factors.	[44]
Efflux pump	*A. baumannii*	AgNPs synthesized using *Acroptilon repens* suppressed the expression of *A. baumannii* efflux pump genes.	[45]
	*A. baumannii*	*Holarrhena antidysenterica* extract and its pure compound, conessine interfere with AdeIJK pump in *A. baumannii*.	[46]
	*P. aeruginosa*	Conessine inhibited MexAB-OprM efflux pump in *P. aeruginosa.*	[47]
	*P. aeruginosa*	Berberine inhibited aminoglycoside-resistant *P. aeruginosa* through inhibition of MexXY multidrug efflux pump.	[48]

*P. aeruginosa=Pseudomonas aeruginosa, E. coli=Escherichia coli, A. baumannii=Acinetobacter baumannii,* St×2 = Shiga toxin 2, *C. difficile=Clostridium difficile*, *V. cholera*=*Vibrio cholera*

An extract of *Fructus mume* inhibited the mucoviscosity and capsular polysaccharides in *K. pneumoniae* through downregulation of *cps* [41]. *Fructus mume* extract and citric acid, an important organic acid from plants, have been reported to suppress the expression of mRNA levels of *cps* biosynthesis genes in *K. pneumoniae* [41]. The capsule plays a crucial role against phagocytosis by preventing complement disposition. A bioactive compound named ASK2 isolated from *Streptomyces* spp. acted as an opsonin, resulting in increased phagocytosis by RAW264.7 and J774.A.1 macrophages [50].

Rhodomyrtone inhibits two important enzymes: glycosyltransferase and UTP-glucose-1-phosphate uridylyltransferase and three metabolites: UDP-glucose, UDP-glucuronic acid, and UDP-*N*-acetyl-D-galactosamine, which are involved in the biosynthesis of the *S. pneumoniae* capsule [39]. Rhodomyrtone-treated *S. pneumoniae* had significantly fewer capsules than the controls [39]. Furthermore, rhodomyrtone treatment increased phagocytosis of *S. pneumoniae* cells by RAW264.7 macrophages [38].

Carvacrol, a pure compound isolated from oregano oil, inhibited the production of *C. difficile* toxin through the downregulation of the toxin genes *tcdA* and *tcdB* [42]. Crude polyphenol extracted from immature apples inhibited fluid accumulation induced by *Vibrio cholerae* toxin in mice. The extract inhibited ADP-ribosyltransferase activity, resulting in reduced fluid accumulation in mice diarrhea models [43]. AHU3 suppressed the expression of the Shiga toxin (Stx2) in *E. coli* O157:H7. The compound inhibited the expression of RecA, resulting in the suppression of prophage genes that encode virulence factors, including Stx2 [44].

Silver nanoparticles synthesized using *Acroptilon repens* suppressed the expression of *A. baumannii* efflux pump genes, including *AdeA*, *AdeC*, *AdeS*, *AdeR*, *AdeI*, *AdeJ*, and *AdeK* [45]. *Holarrhena antidysenterica* extract and its pure compound (conessin) interfered with the AdeIJK pump in *A. baumannii* [46]. Conessine inhibited the MexAB-OprM efflux pump in *P. aeruginosa* [47]. The extract and conessine act as resistance modifying agents to restore the activity of novobiocin and rifampicin against *A. baumannii*, which are extensively drug-resistant [46]. Conessine significantly decreased the minimum inhibitory concentration (MIC) of antibiotics by at least 8-fold in the overexpressed MexAB-OprM strain of *P. aeruginosa* [47]. This mechanism of action was further confirmed by evaluating the MIC value of antibiotics against the mutant *P. aeruginosa* MexB. The synergistic effects of antibiotics, including cefotaxime, erythromycin, and rifampicin, as well as conessine, against the mutant, have been demonstrated [47]. Berberine inhibited aminoglycoside-resistant *P. aeruginosa* through inhibition of the MexXY multidrug efflux pump [48]. Furthermore, the pure compound exhibited a synergistic activity along with aminoglycoside antibiotics, including amikacin and piperacillin, against multidrug-resistant *P. aeruginosa* [48].

## Anti-Virulence Factors based on Anti-Quorum Sensing Activity

The widespread use of antimicrobial agents is the main driving force behind the evolution of antibiotic resistance. Conventional treatment of infection is based on antibiotics that kill pathogens. To overcome infections caused by antibiotic-resistant bacteria, the anti-virulence properties of natural products, based on quorum sensing, can be used for treatment. Bacterial quorum sensing systems regulate phenotype expression, including virulence factors. Inhibition of quorum sensing does not inhibit bacterial growth. Therefore, inhibition of bacterial virulence factors by inhibition of quorum sensing may be an alternative strategy to overcome antibiotic resistance.

Quorum quenching is a strategy that disrupts bacterial communication. Hence, quorum quenching compounds can inhibit the production of bacterial virulence factors [51]. There are four possible mechanisms by which quorum quenching compounds inhibit bacterial communication [51]: (i) inhibition of the synthesis of autoinducer molecules, (ii) inactivation of autoinducer molecules or digestion of molecules, (iii) competition of binding between autoinducers and receptors, and (iv) blocking the autoinducer-receptor complex.

As shown in Table-2 [49, 52–58], a *Delftia tsuruhatensis* extract inhibited biofilm, motility, elastase activity, and protease activity in *P. aeruginosa*. The extract suppressed the expression of the quorum sensing genes *LasI*, *LasR*, *RhlI*, and *RhlR*. In *C*. *violaceum*, which is a biomonitor strain, the extract completely inhibited the production of violacein [52]. The ethyl acetate extract of *Blastobotrys parvus* PPR3 exhibited anti-virulence factors, including biofilm, elastase, and phytocyanin production; motility; and swarming based on the anti-quorum sensing activity of *P. aeruginosa* [53]. *Amphipterygium adstringens* extract and its isolated compound, a mixture of anacardic acid, showed anti-quorum sensing activity against the production of violacein and inhibition of pyocyanin, as well as production of rhamnolipid, in *P. aeruginosa* [54]. An extract of *Forsythia suspensa* exhibited anti-quorum sensing activity against *C. violaceum* by inhibiting the CviR receptor, resulting in reduced biofilm formation [55]. However, the extract did not inhibit the synthesis or degradation of the autoinducer [55]. In *C. violaceum*, cyanidin inhibited violacein production, as well as reduced biofilm formation and production of exopolysaccharide, in *K. pneumoniae* [56]. In addition, a combination of erythromycin and tetracycline with cyanidin has been reported to have synergistic effects against *K. pneumoniae* [56]. An extract of *R. tomentosa* reduced biofilm formation of *S. pyogenes* through inhibition of quorum sensing [49]. Furthermore, the extract and pure compound, rhodomyrtone, acted as biofilm inhibitors against *S. aureus*, *Staphylococcus epidermidis* [59], and *S. pneumoniae* [38]. In addition, rhodomyrtone suppressed the expression of *S. pneumoniae* arginine deiminase [39], resulting in the inhibition of biofilm maturation [38]. Cannabigerol inhibited bioluminescence regulated by quorum sensing and the formation of *Vibrio harveyi* biofilm through downregulation of *LuxR* [57]. Furthermore, the pure compound interfered with the transmission of autoinducer signals in *V. harveyi* [57]. *Chlamydomonas reinhardii* secretes compounds that mimic the action of *N*-acyl-l-homoserine lactone as autoinducers. The secreted compounds could stimulate CepR or LasR, resulting in the inhibition of quorum sensing in bacterial quorum sensing reporter strains [57]. *Origanum vulgare* subsp. *Hirtum* and *Rosmarinus officinalis* extracts were reported to inhibit *E. coli* biofilm formation and swarming and swimming motilities. The extract also inhibited autoinducer-2 signaling in *V. harveyi*.

**Table-2:** Activity of bioactive compounds from the plants against the bacterial virulence factors based on anti-quorum sensing activity.

Bioactive compounds	Microorganisms	Activity	Reference
*Delftia tsuruhatensis* extract	*P. aeruginosa*	The extract inhibited biofilm, elastase activity, and protease activity through suppression of quorum sensing regulatory genes.	[52]
*Blastobotrys parvus* PPR3 extract	*P. aeruginosa*	The extract inhibited biofilm, elastase production, and phytocyanin, motility, and swarming.	[53]
*Amphipterygium adstringens* extract and AAM	*P. aeruginosa* *C. violaceum*	The extract showed anti-quorum sensing activity against *C. violaceum* and inhibition of pyocyanin, rhamnolipid production in *P. aeruginosa*.	[54]
*Forsythia suspense* extract	*C. violaceum*	The extract exhibited anti-quorum sensing activity against *C. violaceum* by inhibition of CviR receptor.	[55]
Cyanidin	*K. pneumoniae* *C. violaceum*	Cyanidin inhibited the production of violacein in *C. violaceum* and reduced the formation of biofilm, and the production of exopolysaccharide in *K. pneumoniae.*	[56]
*Rhodomyrtus tomentosa* extract	*S. pyogenes* *C. violaceum*	The extract reduced *S. pyogenes* biofilm through quorum sensing inhibition as observed in *C. violaceum*.	[49]
Cannabigerol	*V. harveyi*	Cannabigerol inhibited quorum sensing regulated bioluminescence and the formation of biofilm in *V. harveyi* through down regulation of *LuxR* gene.	[57]
*Chlamydomonas reinhardtii*	*C. violaceum* *Pseudomonas putida* *V. harveyi*	*Chlamydomonas reinhardtii* secreted compounds that mimic the activity of autoinducers.	
*Origanum vulgare* subsp*.* *hirtum* extract	*E. coli* *V. harveyi*	The extract inhibited the biofilm formation and the swarming and swimming motilities of *E. coli*. The extract possessed autoinducer-2 signaling activity against *V. harveyi*.	[58]
*Rosmarinus officinalis*	*E. coli* *V. harveyi*	The extract inhibited *E. coli* biofilm formation and the swarming and swimming motilities. The extract also inhibited autoinducer-2 signaling in *V. harveyi*.	[58]

AAM=Anacardic acid mixture, *P. aeruginosa*=*Pseudomonas aeruginosa*, *V. harveyi*=*Vibrio harveyi*, *E. coli*=*Escherichia coli*, *C. violaceum*=*Chromobacterium violaceum*, *S. pneumoniae*=*Streptococcus pneumoniae*, *K. pneumoniae*=*Klebsiella pneumoniae*, *S. pyogenes*=*Streptococcus pyogenes*

## Bacterial Clearance

Bacterial virulence factors play a role in escaping the host’s immune response. For example, the *S. pneumoniae* polysaccharide capsule plays a role in phagocytosis by preventing complement disposition [17]. Similarly, the deposition of C3b and the binding of C-reactive protein and C1q to the biofilm of *S. pneumoniae* were impaired, compared with planktonic cells [12]. Efflux pumps in many bacteria increase the MIC values of antibiotics against pathogens. Therefore, much research has focused on inhibiting bacterial virulence factors based on inhibiting quorum sensing. Ideally, the compounds must inhibit virulence factors and enhance the activities of the host’s immune cells. Furthermore, the compounds must act as resistant modifying agents. Combined with antibiotics, these agents inhibit bacterial resistance and restore antibiotic activity.

Rhodomyrtone inhibited biofilm formation and established biofilm formation in *S. pneumoniae* [38]. The expression of arginine deiminase, which is suppressed by rhodomyrtone [39], inhibited biofilm maturation in organisms [37]. Rhodomyrtone inhibited two enzymes: glycosyltransferase and UTP-glucose-1-phosphate uridylyltransferase and three metabolites: UDP-*N*-acetyl-D-galactosamine, UDP-glucose, and UDP-glucuronic acid, which are involved in the biosynthesis of the *S. pneumoniae* capsule [39]. Rhodomyrtone-treated *S. pneumoniae* had remarkably fewer capsules than the control [39]. Furthermore, phagocytosis of *S. pneumoniae* cells by RAW264.7 macrophages increased after treatment with rhodomyrtone [38]. Therefore, the bacteria lost the virulence factors that could be removed by the host’s immune system.

Conessine inhibited the MexAB-OprM efflux pump in *P. aeruginosa* [47]. The pure compound acted as a resistance modifying agent to restore the activity of novobiocin and rifampicin against extensively drug-resistant *A. baumannii* [46]. Conessine significantly decreased the MIC of antibiotics by at least 8-fold in the MexAB-OprM-overexpressing strain of *P. aeruginosa* [47]. This mechanism of action was confirmed by investigating the MIC values of antibiotics against the *P. aeruginosa* MexB mutant. The synergistic effects of antibiotics, including cefotaxime, erythromycin, and rifampicin, along with conessine against the mutant, have been demonstrated [47]. Therefore, these compounds act as resistance modifying agents to restore antibiotic activity against multidrug-resistant bacteria.

Synthesis of nanoparticles using plant extracts or bioactive compounds has been reported as one option to improve the efficacy of the compounds that inhibit bacterial virulence factors. Biofilm formation produced by *P. aeruginosa* was completely inhibited by treatment with silver nanoparticles synthesized using *Glochidion lanceolarium* extract [60]. Similarly, copper nanoparticles synthesized using *Cardiospermum halicacabum* leaf extract inhibited the development of *P. aeruginosa* biofilms [61]. The nanoparticles could attach to the bacterial cells, disrupting the bacterial cell wall or cell membrane to inhibit biofilm formation [58, 61].

## Conclusion

Bacterial pathogenesis is carried out by bacterial virulence factors that are regulated by the bacterial quorum sensing system. The inhibition of bacterial virulence factors based on the inhibition of quorum sensing activity is a new alternative to inhibit pathogenic bacteria without antibiotic resistance. Naturally-derived compounds act as anti-virulence factors and anti-quorum sensing agents against pathogenic bacteria. Bacteria that have lost virulence factors that can be removed by the host’s immune system. In addition, the compounds act as resistance modifying agents to restore antibiotic activity against multidrug-resistant bacteria. Therefore, anti-virulence factors of naturally-derived compounds can be used for treating bacterial infections.

## Authors’ Contributions

WK, CNS, VN, MLP, and WM: Conceived and designed the study. WK, VN, SS, AKP, and WM: Conducted the literature review and prepared the tables and figures. WK, SS, AKP, and WM: Drafted the manuscript. CNS, VN, and MLP: Critically revised the manuscript for important intellectual content. All authors have read, reviewed, and approved the final manuscript.
